# Adaptive Total Variation Minimization-Based Image Enhancement from Flash and No-Flash Pairs

**DOI:** 10.1155/2014/319506

**Published:** 2014-03-04

**Authors:** Sang Min Yoon, Yeon Ju Lee, Gang-Joon Yoon, Jungho Yoon

**Affiliations:** ^1^School of Computer Science, Kookmin University, 77 Jeongneung-ro, Seogbuk-gu, Seoul 136-702, Republic of Korea; ^2^Institute of Mathematical Sciences, Ewha Womans University, 52 Ewhayeodae-gil, Seodaemun-gu, Seoul 120-750, Republic of Korea; ^3^Department of Mathematics, Ewha Womans University, 52 Ewhayeodae-gil, Seodaemun-gu, Seoul 120-750, Republic of Korea

## Abstract

We present a novel approach for enhancing the quality of an image captured from a pair of flash and no-flash images. The main idea for image enhancement is to generate a new image by combining the ambient light of the no-flash image and the details of the flash image. In this approach, we propose a method based on Adaptive Total Variation Minimization (ATVM) so that it has an efficient image denoising effect by preserving strong gradients of the flash image. Some numerical results are presented to demonstrate the effectiveness of the proposed scheme.

## 1. Introduction

A camera flash provides a practical and tractable lighting environment creating vivid and bright images. Yet, the camera flash produces some undesirable effects like uneven lighting and distracting sharp shadows [1]. To solve the dilemma, a flash photography method has been attracting great interest [1–5] to transfer the details from the flash image, while removing the ambient shadows captured from the no-flash image.

We briefly address the method of generating a high-quality image from two captured images: a flash image (*Z*) and a no-flash image (*Y*). The task in hand is to generate a new image (*X*) that contains the ambient light of the no-flash image and preserves the details of the flash image. To be more precise, since the given no-flash image *Y* might be noisy or blurry, we generate an estimated version $\hat{Y}$ of *Y*, enhanced with the help of the flash image. Also, we produce a filtered version $\hat{Z}$ from the flash image *Z* so that $Z-\hat{Z}$ can provide details. Then, a new image is generated by the two images called a basis layer and a detail layer, respectively, as follows [1]:

(1) $$\begin{array}{c} X=\hat{Y}+\tau \left(Z-\hat{Z}\right). \end{array}$$

Here *τ* is a parameter controlling the tradeoff.

The main issue in this method is how to generate the two images $\hat{Y}$ and $\hat{Z}$. Recently, numerous approaches for image denoising and enhancement from flash effects have been proposed by modeling the behavior of flash light or understanding the geometry of the scene. In order to extract $\hat{Y}$ and $\hat{Z}$ for image denosing, some techniques based on filtering method have been proposed. The bilateral filtering technique by Tomasi and Manduchi [4] and the total variation (TV) minimization technique by Wang et al. [6] have used edge-preserving and noise-reducing smoothing filters. Eisemann and Durand [5] proposed a cross-bilateral filter where they modified the bilateral filter [4] and computed the edge-preserving term as a function of the flash-image values. Their method preserves edges that do not really appear in the no-flash image. Eisemann and Durand [5] replace the intensity value at each pixel in an image by a weighted average of intensity values from nearby pixels.

PDE-based denoising methods have been used for image smoothing with edge preservation, which are based on variational approach of energy functional minimization. A popular variational denoising method is the TV minimizing process of Rudin-Osher-Fatemi [7]. In the literature of image restoration, the TV regularization term has an effect of preserving salient edges and removing noises. When the variation minimizing influence is too strong, however, it causes smooth regions to be flat or constant so that the restored image looks unnatural. This is known as the staircasing effect [8], which is mainly due to the fact that the TV minimization method approximates the image to piecewise constant approximation. To avoid the staircasing effect and obtain a higher order approximation of the reconstructed image, several variants of the TV functional have been proposed [8–10].

In this work, we especially employ an adapted TV minimization (ATVM) technique [11] with high accuracy so that it can preserve the details of flash image. To preserve strong edges while smoothing noises, we add the TV regularization term to the moving least squares method with the weight functions that consider the similarity of the local areas between the evaluation and the reference positions. In our proposed method, we construct a local polynomial approximation *p* by solving the TV based minimization at each pixel position **x** as follows:

(2) f(x)∶=argminp∈ΠL⁡{∑n=1N|∇p(xn)|+μ2|p(xn)−I(xn)|2× θ(||x−xn||)},

where ∇*g* = (∂*g*/∂*x*, ∂*g*/∂*y*) indicates the gradient vector of *g*, Π_*L*_ is the space of bivariate polynomials of degree ≤*L*, and *θ* is a weighting function. The adaptive TV minimization scheme retains the merit of the original method which preserves the geometrical information and performs the image denoising, while obtaining a solution image of high accuracy.

## 2. Our Proposed Approach

Suppose that our observed data is a discrete sampling of a function at a point set in a domain *𝒟* ⊂ ℝ^2^. That is, an image *I* is regarded as a set of function values *I* : = {*I*(*i*, *j*) : *i* = 1,…, *M*_1_, *j* = 1,…, *M*_2_}. In this paper, we use the notation [1,…, *M*_1_]×[1,…, *M*_2_] = (**x**_1_, **x**_2_,…, **x**_*N*_), where *N* = *M*_1_ × *M*_2_ is the size of the image. In most cases, images are contaminated by the noise introduced by the image acquisition process. So, an image can be written as *I*(**x**_*n*_) = *f*(**x**_*n*_) + *ϵ*_*n*_, *n* = 1,…, *N*, where *f*(**x**_*n*_) is the value of an underlying function *f* and *ϵ*_*n*_ indicates the additive noise at the location **x**_*n*_. The denoising method to construct a new image from no-flash and flash images is discussed below.

*(i) Denoising Method*. In this section, we propose a new energy functional to be minimized for image denoising which is useful for flash photography. Given a non-flash image *Y* and a flash image *Z*, our image denoising method to extract $\hat{Y}$ and $\hat{Z}$ is designed to generate a new high-quality image defined on the domain *Ω*. Since flash and non-flash images have different features, it is desirable to construct a denoising scheme considering the characteristics of both images.

For a given noisy image *I*, the TV minimization technique generates a denoised image $\hat{I}$ by solving the following minimization problem:

(3) $$\begin{array}{c} \hat{I}=\underset{u}{\text{argmin}}\int_{𝒟}{\left|u-I\right|}^{2}dx+\lambda \int_{𝒟}\left|\nabla u\right|dx, \end{array}$$

where **x** denotes the position vector of the pixel and ∇*u* is the gradient operator. This model is particularly useful for flash photography since the minimization of the TV norm eliminates small scaled structures in images very fast from the image. The incorporation of the TV minimization term reduces the influence of outliers which is mainly due to the uneven lighting. The second term in (3) is called the total variation norm, and its minimizing has the property of preserving sharp discontinuities (edges) in images while removing noise. This is a desirable property for images, since the visual quality of an image greatly depends on the preservation of edges. However, this TV scheme processes the observed image toward a piecewise constant image, which exhibits many false jump discontinuities and is visually unpleasant. This is mainly due to the fact that the TV minimization variation method approximates to an image with a first-order accuracy. In this case, it may lose the specific features of flash images. In this point of view, we employ an adapted TV minimization technique which can preserve the details of flash image [11]. Let *I* be a given reference image defined on a domain *Ω*, and let **x** be a fixed point in *Ω*. We seek a solution by using local polynomial of degree *L* in ℝ^2^ : *p*(**r**): = *p*_**x**_(**r**)∶ = ∑_|*α*|_1_≤*L*_ *c*_*α*_ **r** ^*α*^. The coefficients *c*_*α*_ are obtained by minimizing the following energy functional:

(4) $$\begin{array}{c} \underset{p\in \Pi_{L}}{\text{argmin}}\left\{\sum_{n=1}^{N}\left|\nabla p\left(x_{n}\right)\right|+\frac{\mu }{2}{\left|p\left(x_{n}\right)-I\left(x_{n}\right)\right|}^{2}\theta \left(x,x_{n}\right)\right\}, \end{array}$$

where Π_*L*_ is the space of bivariate polynomials of degree ≤*L* and *θ* is a weighting function. In this construction, we use a weight function *θ* defined as

(5) $$\begin{array}{c} \theta \left(x,r\right)=\exp\left\{-\frac{G_{a}*\left({||I\left(x+\cdot \right)-I\left(r+\cdot \right)||}^{2}\right)\left(0\right)}{h_{0}^{2}}\right\}, \end{array}$$

where *h*_0_ is a small positive value, *G*_*a*_ is the Gaussian function with standard deviation *a*, and +· denotes the shifting operator. The weight function is data adaptive and considers the similarity of the local areas between two positions **x** and **r**. In our proposed method, we construct *p* at each pixel in the image, and so we are solving the minimization problem in (4) locally at each pixel in *Ω*. Then, the overall approximation function $\hat{I}$ becomes $\hat{I}(x):=p(x):=p_{x}(x)$ for all **x** ∈ *Ω*.

For solving our proposed minimization model (4), we employ the split Bregman iteration algorithm [12]. By placing the iterative minimization scheme into our problem (4), we get the following iteration steps for each **x**:

(6) (a) pk+1(x)=min⁡p{∑n=1Nμ2|p(xn)−I(xn)|2θ(x,xn)+λ2×|dk(xn)−∇p(xn)−bk(xn)|2× θ(x,xn)}(b) dk+1(x)=min⁡d|d|+λ2|d−∇pk+1(x)−bk(x)|2=shrink(∇pk+1(x)+bk(x),1λ)(c) bk+1(x)=bk(x)+∇pk+1(x)−dk+1(x).

Without the first term in (4), the energy functional in (4) becomes just as that used in the conventional least square approximation which fits data by local polynomial approximation [13–15]. In our scheme (4), the TV regularization term endows the better denoising property. Normally, a least square approximation is weak against noise, since, in general, least squares methods are weak against outliers. On the other hand, TV based methods are strong against outliers, since outliers have large variation values. The TV regularization term eliminates noise very fast and helps the regularized least squares framework to produce a better approximation to the original noise-free image. Therefore, the proposed scheme can also provide a better solution than conventional TV schemes.

*(ii) General Algorithm*. Let *Y* and *Z* be the non-flash and the flash images, respectively. The algorithm to construct a new image *X* is given as follows.

**Algorithm 1**

*Step  1*. Apply the ATVM algorithm to generate denoised images $\hat{Y}$ and $\hat{Z}$ from the given images non-flash image *Y* and the flash image *Z*.

*Step  2*. Choose a suitable tension parameter *τ* (We use *τ* = 0.02 for the all experiments).

*Step  3*. Construct a new image by using the following equation

(7) $$\begin{array}{c} X=\hat{Y}+\tau \left(Z-\hat{Z}\right). \end{array}$$

## 3. Experiments

In this section, we conduct experimental results on two flash and no-flash image pairs to show the effectiveness of our proposed flash photography with a two-step approach.

**Figure 1:**
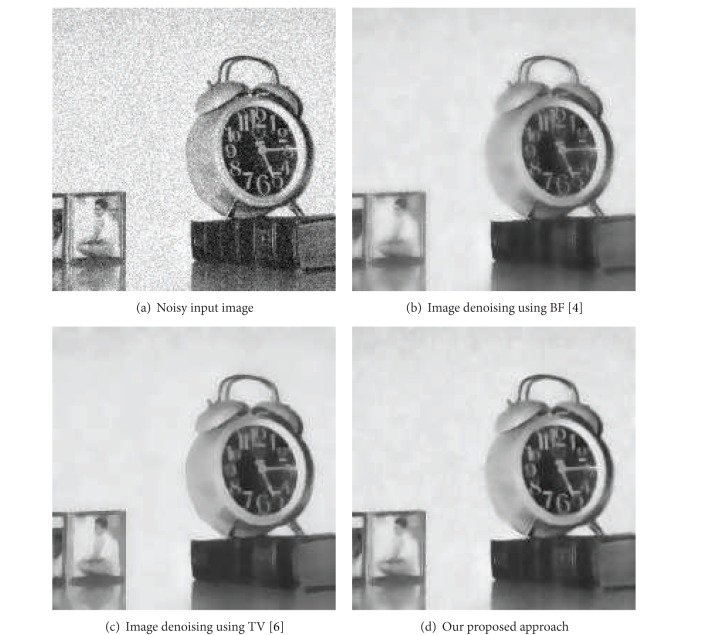
Comparison of image denoising between previous approaches and our proposed approach.

First, we need to efficiently denoise the no-flash image while transferring the fine detail of the flash image and maintaining the ambient lighting of the no-flash image. ATVM based denoising method yields much better results than remarkable existing approaches like bilateral filter and traditional TV.  Figure 1 provides the comparison that our proposed ATVM method is very powerful to remove the noise while keeping the details of the target object. For detailed comparison of image denoising, additive white Gaussian noise (AWGN) with *σ* = 0.6 is added as shown in Figure 1(a). We can remove the noise by using bilateral filter in Figure 1(b), traditional TV in Figure 1(c), and our proposed approach in Figure 1(d). Our proposed approach was better than the previous approaches in emphasizing the details and edges of the target object. This characteristic is very effective to transfer the details of the target object from that captured from the flash image. The comparison of image denoising and recovering as shown in Figure 1 shows that our proposed ATVM (in Figure 1(d)) also removes the staircasing effect within the object.  Table 1 shows a quantitative comparison of image denoising and deblurring using Peak Signal-to-Noise Ratio (PSNR) values between our result and the previous approaches.  Table 1 proves that our proposed ATVM method out performs the previous approaches directly by transferring the details while removing the ambient shadows.

**Figure 2:**
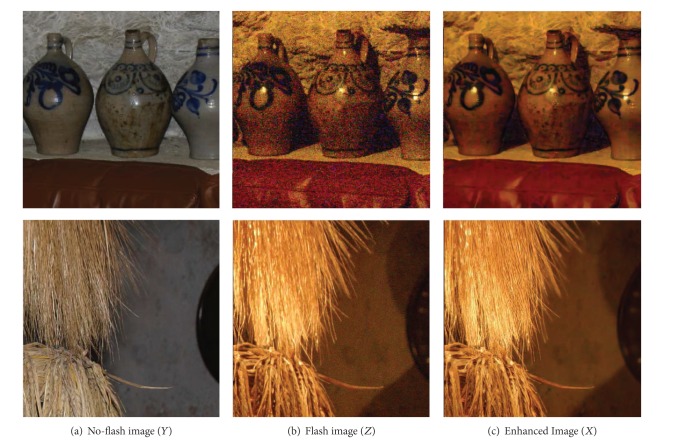
Image enhancement from real flash/no-flash image pairs.

The second step in our experiments is to create a new image from denoised flash and no-flash image pair using (1). Based on the experimental results using ATVM, we can dramatically improve the quality of the image as shown in Figure 2. It shows the enhanced image using real flash and no-flash image pairs as represented in Figures 2(a) and 2(b). The noise from no-flash image as shown in Figure 2(b) is significantly reduced, but the ambient lighting in no-flash image in Figure 2(a) is still kept in our proposed approach.  Figure 2(c) also shows that our proposed approach preserves both details of the object and smooth continuity between foreground and background even though the borders of the foreground and background are not clearly separated.

## 4. Conclusion

In this work, we propose a novel image enhancement approach from flash and no-flash image pairs. To combine the strengths of the flash and no-flash images, we adopt the MLS method and the TV minimization together. The MLS method performs fast denoising and enables us to find a sufficiently smooth approximation. And the TV based image denoising and debluring approach shows robustness in reducing the noise while keeping the edges from flash/no-flash pairs. The experiments using real images show that ATVM based image enhancement answers our intention considerably. Enhancing the resolution from the flash and no-flash pairs, the proposed scheme retains the merit of the original TV minimization method which preserves the geometrical information while obtaining an image of higher order approximation. In particular, our proposed ATVM works well on maintaining the detail of the target object while reducing the noise without prior information.

## Figures and Tables

**Table 1:** Quantitative comparison of image denoising between our approach and previous approaches using PSNR.

	Noisy image	BF [4] approach	TV [6] approach	Our approach
PSNR	24.6150	27.5591	27.4700	29.8629
